# Circular RNAs as Novel Diagnostic Biomarkers and Therapeutic Targets in Kidney Disease

**DOI:** 10.3389/fmed.2021.714958

**Published:** 2021-09-16

**Authors:** Jianwen Yu, Danli Xie, Naya Huang, Qin Zhou

**Affiliations:** ^1^Department of Nephrology, The First Affiliated Hospital, Sun Yat-sen University, Guangzhou, China; ^2^National Health Commission Key Laboratory of Nephrology, The First Affiliated Hospital, Sun Yat-sen University, Guangzhou, China; ^3^Guangdong Provincial Key Laboratory of Nephrology, The First Affiliated Hospital, Sun Yat-sen University, Guangzhou, China; ^4^Department of Nephrology, Shishi General Hospital, Quanzhou, China

**Keywords:** circular RNAs, biogenesis, biological functions, biomarkers, therapeutic targets, kidney diseases

## Abstract

Circular RNAs (circRNAs) are a novel type of non-coding RNAs that have aroused growing attention in this decade. They are widely expressed in eukaryotes and generally have high stability owing to their special closed-loop structure. Many circRNAs are abundant, evolutionarily conserved, and exhibit cell-type-specific and tissue-specific expression patterns. Mounting evidence suggests that circRNAs have regulatory potency for gene expression by acting as microRNA sponges, interacting with proteins, regulating transcription, or directly undergoing translation. Dysregulated expression of circRNAs were found in many pathological conditions and contribute to the pathogenesis and progression of various disorders, including renal diseases. Recent studies have revealed that circRNAs may serve as novel reliable biomarkers for the diagnosis and prognosis prediction of multiple kidney diseases, such as renal cell carcinoma (RCC), acute kidney injury (AKI), diabetic kidney disease (DKD), and other glomerular diseases. Furthermore, circRNAs expressed by intrinsic kidney cells are shown to play a substantial role in kidney injury, mostly reported in DKD and RCC. Herein, we review the biogenesis and biological functions of circRNAs, and summarize their roles as promising biomarkers and therapeutic targets in common kidney diseases.

## Introduction

Circular RNAs (circRNAs) are a new type of non-coding RNA molecules that have attracted more and more attention in recent years (1). Although initially they are overlooked as redundant products from mis-splicing events yielding at low expression levels (2), a growing number of studies indicate that circRNAs are widely expressed in eukaryotic cells, from fungi, plants, to metazoans, such as fruit fly, mouse as well as human (3–6). Unlike linear RNAs, circRNAs are single stranded and covalently closed RNA transcripts. The structure characteristic renders circRNAs naturally resistant to exonuclease-mediated degradation, thus producing high stability (7). Many circRNAs are abundantly expressed, evolutionarily conserved, and exhibit cell-type-specific, tissue-specific and developmental-stage-specific expression patterns (3, 8–10). CircRNAs are formed by a non-canonical splicing event called back-splicing during which a downstream 5′ splice donor site is covalently joined to an upstream 3′ splice acceptor site (11, 12). They mainly arise from protein-coding exons, but can also from introns, untranslated, or intergenic regions of the genome (12). According to formation modes and sequences, circular RNAs can be classified into three types: exonic circRNA (ecircRNA), which is generated from back-spliced exons (8); intronic circRNA (ciRNA), which arises from intron lariats (13); exon-intron circRNA (EIciRNA), which consists of both exons and introns (14). The biological functions of circRNAs have been extensively investigated in this decade. The most frequently proposed mechanism of action is to act as microRNA (miRNA) sponges (12, 15). Moreover, circRNAs have been shown to function through interacting with proteins, regulating transcription, or directly undergoing translation (1). Nevertheless, the functions of most circRNAs identified to date remain largely elusive.

Since the recent findings that circRNAs are ubiquitous in human tissues and differentially expressed under pathological states, their functional relevance in diseases have been increasingly explored. The majority of researches have been focused on their roles in cancer (16), cardiovascular diseases (17), diabetes mellitus (18), and neurological disorders (19). For example, as a well-characterized circRNA in human diseases, ciRS-7 is initially found to be abundantly expressed in neuronal tissues and participated in neuronal development by acting as a sponge for miR-7 (12, 15). Subsequent studies reveal it could also exert oncogenic functions during tumorigenesis and modulate insulin secretion (20, 21), indicating that circRNAs may play important roles under diverse pathophysiological states. In recent years, there has been an increasing focus on characterizing the roles of circRNAs in kidney diseases, including renal cell carcinoma (RCC), acute kidney injury (AKI), diabetic kidney disease (DKD), and other glomerular diseases (22–24). These studies explored the feasibility of circRNAs as non-invasive biomarkers for the diagnosis and outcome prediction of specific kidney diseases. Furthermore, the functions of circRNAs expressed by kidney resident cells in the pathogenesis and progression of renal disorders have also been actively investigated. This review will introduce the biogenesis and biological function of circRNAs, and focus on state-of-art regarding circRNAs as novel biomarkers and therapeutic targets in common kidney diseases.

## Biogenesis and Properties of CircRNAs

### Biogenesis and Regulation of CircRNAs

**Figure 1:**
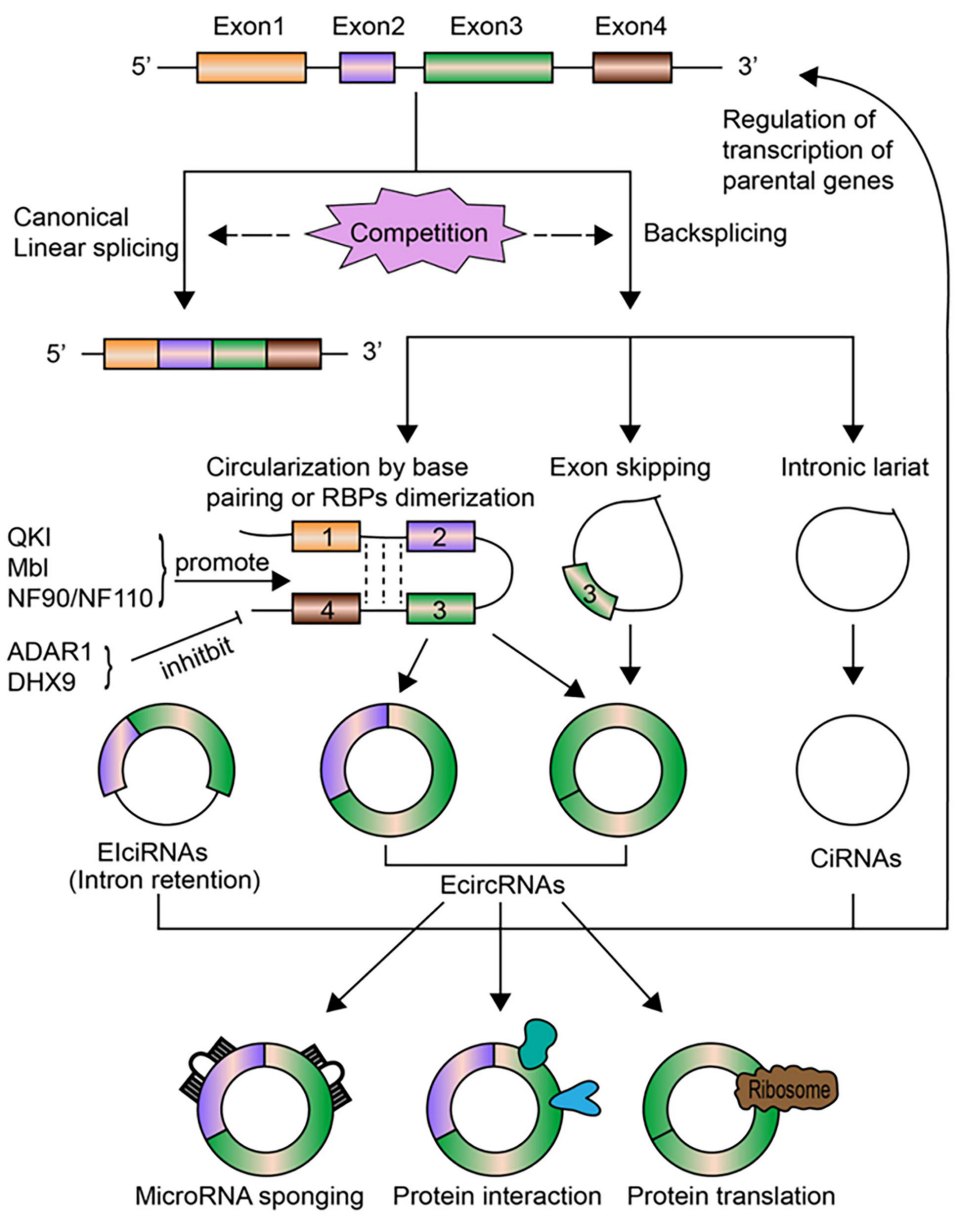
The biogenesis, regulation, and functions of circRNAs. CircRNAs are transcribed from pre-mRNAs through the non-canonical back-splicing event. The back-splicing process involves different mechanisms, including circularization by base paring of intronic sequences or RBPs dimerization, exon skipping, and intronic lariat, which generates three types of circRNAs: ecircRNAs, ciRNAs, and EIciRNAs. Some molecules, including QKI, Mbl, NF90/NF110, positively regulate the biogenesis of circRNAs, while ADAR1 and DHX9 inhibit circRNAs formation. CircRNA biogenesis *per se* competes with canonical linear splicing. The most well-studied functions of circRNAs is to act as miRNA sponges to modulate the expression of miRNA target genes. CircRNAs can bind to RNA pol II or other RBPs to affect transcription levels of their parental genes, especially for nuclear-retained ciRNAs and EIciRNAs. CircRNAs can act as sponges, decoys, scaffolds for proteins or regulate protein trafficking through interacting with specific proteins. Certain circRNAs can be translated into proteins under stress circumstances.

CircRNAs are transcribed from pre-mRNAs through a non-canonical splicing event called back-splicing (Figure 1), which is regarded as a type of alternative splicing by a broad definition. In general, during a back-splicing event, a downstream 5′ splice donor site is linked to an upstream 3′ splice acceptor site via covalent bonds to form a closed-loop circRNA. Though circRNA circularization and linear splicing compete against each other, mutagenesis analyses in circRNA expression vectors as well as blocking experiments using the inhibitor for spliceosome assembly have shown that both canonical splice sites and spliceosomal machinery are required for circRNA biogenesis (25, 26). The processing of back-splicing circularization has not been fully elucidated, and several working models have been proposed (Figure 1). In the first model, the direct base paring between inverted repeat elements in the flanking intron sequences (such as Alu elements) brings a downstream splice donor site into close proximity with an upstream acceptor site, which may facilitate back-splicing looping by the canonical splicing machinery (27). RNA binding proteins (RBPs) including Quaking (QKI) and fused in sarcoma (FUS) could also drive exon circularization in a similar manner by dimerization of RBPs that bind to specific motifs in the flanking introns (28). These two proposed models lead to the formation of ecircRNAs or EIciRNAs (circRNAs with retained introns). Other hypothesis includes the exon-skipping model, in which alternative exons are spliced out to form exon lariat that end up as ecircRNAs by internal back-splicing (29). Last but not the least, intronic lariat precursors that escape from the debranching step of canonical linear splicing can result in the production of ciRNAs (13). Following biogenesis, ecircRNAs are normally translocated into the cytoplasm, while ciRNAs and EIciRNAs are predominately retained in the nucleus.

The regulation of circRNAs biogenesis is crucial for characterizing their functional roles under pathophysiological conditions, but is still largely unclear. Emerging lines of evidence have identified that several regulatory mediators are involved in circRNAs formation (Figure 1). As mentioned above, QKI was found to promote circRNAs production during human epithelial–mesenchymal transition (EMT) (28). QKI could bind to specific motifs in introns flanking circularized exons and bring these exons closer together via dimerization, which facilitates circularization and results in enhanced circRNAs formation. Overexpression of the splicing factor Muscleblind (Mbl), or the insertion of synthetic Mbl-binding sites into the introns flanking circRNA-forming exons in an expression vector, promoted exon circularization, suggesting that Mbl can enhance circRNAs formation in a similar way (25). Adenosine deaminase 1 acting on RNA (ADAR1) has been found to suppress circRNAs biogenesis by disrupting the looping of flanking introns relying on base pairing between inverted repeats. Knockdown of ADAR1 promoted the up-regulation of certain circRNAs (10). ATP-dependent RNA helicase A (also known as DHX9) has also been shown to negatively regulate circRNAs expression via a similar mechanism (30), while immune factors NF90/NF110 can promote circRNAs production by stabilizing intronic RNA pairs in viral infection (31). The elimination and degradation of circRNAs undoubtedly play a role in their expression levels, yet remains poorly characterized to date. Future studies are warranted to uncover the turnover process of circRNAs.

### The Properties of CircRNAs

One prominent characteristic of circRNAs is that they are strikingly more stable than other types of RNAs, including linear mRNAs and other non-coding RNAs. One study compared the half-lives of four ecircRNAs and their associated linear transcripts and found the ecircRNAs consistently exhibited long half-lives exceeding 48 h in contrast to linear counterparts with half-lives <20 h (8). Another study calculated the half-lives of 60 circRNAs and their linear isoforms in mammary cells and concluded that the median half-life of circRNAs was at least 2.5 times longer than that of linear mRNAs (32). The high stability of circRNAs is mainly attributed to their special closed-loop structures which are resistant to exonuclease-mediated degradation, yet the precise structural mediators involved remain to be investigated. Though circRNAs were initially overlooked as “junk” byproducts of transcription with low expression levels, emerging evidence suggests that circRNAs are widely expressed in a variety of eukaryotic cells and most human tissues (4, 11). As for the abundance of circRNAs, some studies showed they were weakly expressed in certain cell types (32), while others demonstrated the abundance of certain circRNAs was comparable to or considerably higher than that of associated linear counterparts (8, 10, 11). The discrepancy among studies regarding the expression abundance of circRNAs might be due to their cell-type-specific or tissue-specific expression patterns. A striking example is the mouse circRNA circRims2, which is expressed substantially higher than its linear mRNA in mouse adult brain, but is weakly expressed in other mouse tissues (10). Furthermore, many circRNAs are well-conserved in expression and sequence among species (8, 10). Highly expressed circRNAs are more likely to be conserved, indicating that they are functionally important. These characteristics make circRNAs as promising biomarker candidates for human diseases.

## Biological Functions of CircRNAs

The biological functions of circRNAs have been extensively investigated in recent decade. Similar to miRNAs and long non-coding RNAs (lncRNAs), circRNAs have also been found to play an important role in the regulation of gene expression. They may modulate gene expression by distinct modes of action at transcriptional or post-transcriptional levels. The most well-characterized mechanism is to act as sponges for miRNAs [(12, 15); Figure 1]. Meanwhile, circRNAs could affect splicing and regulate transcription levels (14, 25), interact with specific proteins and modulate their functions (33, 34), or directly undergo translation under stress conditions [(35, 36); Figure 1].

### CircRNAs Act as MiRNA Sponges

MiRNAs are an important type of small non-coding RNAs that negatively regulate gene expression by binding to the 3′ untranslated regions (3′ UTR) of messenger RNAs (mRNAs). Many studies have found that circRNAs harboring miRNA binding sites could compete with mRNAs for miRNAs binding and function as miRNA sponges, thus indirectly affect gene expression. The first-characterized and most well-known circRNA to support this model is ciRS-7 (circRNA sponge for miR-7), which contains more than 70 conserved binding sites for miR-7 (12, 15). CiRS-7 functions to bind miR-7 and the interaction is conserved among species and several disease models. Introduction of ciRS-7 expression vector *in vitro* produced a substantial reduction of knockdown effect by miR-7 on its targets, while repressing ciRS-7 levels by miR-671 regained the inhibition on targets genes by miR-7, supporting the role of ciRS-7 as a highly functional sponge for miR-7 (15). However, genetic ablation of the ciRS-7 locus in mice led to the down-regulation of miR-7 and promoted the expression of its targets (37), arguing for the functional significance of the ciRS-7/miR-7 interaction *in vivo*. These discrepant data suggest that whether ciRS-7 exerts as a sponge for miR-7 may depend on specific biological circumstances. In addition to ciRS-7, many other circRNAs have been shown to function as miRNA sponges. The testis-specific circRNA, sex-determining region Y (Sry), which harbors 16 putative target sites for miR-138 in mouse, was demonstrated to serve as a sponge for miR-138 *in vitro* (15). Transcriptomic analysis revealed that circHIPK3 could sequester a group of miRNAs, including miR-124-3p and miR-338-3p, to regulation β-cell functions (21). Another study showed that circHIPK3 was capable to sponge 9 miRNAs with 18 potential binding sites to regulate cell growth (38). In addition, circCCDC66 was found to exert a novel oncogenic function in colorectal cancer via acting as sponges for a set of miRNAs which target oncogenes (39). Although the mechanism as miRNA sponges has been widely studied, partly due to relatively easy operability, only a minority of circRNAs contain multiple miRNA target sites (13, 40), implying most circRNAs might not function by sponging miRNAs.

### CircRNAs Affect Splicing and Regulate Transcription

CircRNAs are generally produced cotranscriptionally via exon circularization from protein-coding genes (27). It has been shown that circRNAs biogenesis, which is dependent on both canonical splice sites and spliceosomal machinery, competes with pre-mRNA splicing on a global cell level, and then affects the expression of transcribed genes (25). In principle, the more an exon is circularized, the less it will appear in the processed mRNA (29). Therefore, the processing of circRNAs *per se* could regulate gene expression at splicing levels. Furthermore, certain circRNAs could also modulate gene transcription. Unlike ecircRNAs which are mainly localized in the cytoplasm, intron-containing circRNAs including EIciRNAs and ciRNAs are more likely to be retained in the nucleus in human cells (13, 14). These two types of circRNAs have been found to directly participate in transcription regulation. Several abundantly expressed EIciRNAs (including EIciEIF3J and EIciPAIP2) and ciRNAs (including ci-ankrd52) were demonstrated to physically interact with polymerase II complex and promote transcription of their parental genes in *cis* (13, 14). Depleting or knockdown of these circRNAs led to the reduced transcription of their parental genes. Whether additional nuclear-retained circRNAs could regulate transcription in a similar manner or in *trans* remains to be explored.

### CircRNAs Function Through Interaction With Proteins

Increasing studies have found that circRNAs may exert their biological functions by interacting with various proteins. In addition to those nuclear-retained circRNAs binding to polymerase II complex to enhance transcription as mentioned above (13, 14), circRNAs can also act as protein sponges or decoys (25), or as scaffolds to facilitate protein complex formation and reaction (33, 34), or participate in protein trafficking (41). One example of circRNAs as protein sponges was from the study of the splicing factor Muscleblind (MBL) and its circular isoform circMbl (25). MBL could directly promote the biosynthesis of circMbl which was found to contain a binding site for MBL, and a strong interaction between them was confirmed. The cooperative association raises the possibility of an autoregulatory loop in which MBL will decrease the level of its own mRNA by enhancing circMbl production, and then circMbl could sponge out excessive MBL protein by binding to it. Circ-Foxo3 is another circRNA functioning as protein sponges as well as scaffolds for protein complex which could bind to the cell cycle proteins cyclin-dependent kinase 2 (CDK2) and cyclin-dependent kinase inhibitor 1 (CDKA1 or p21), resulting in the formation of a ternary complex and cell cycle arrest (33). Another study found that circ-Foxo3 could bind to both p53 and the mouse double-minute 2 (MDM2). As MDM2 mediates both the ubiquitylation of p53 and FOXO3 protein, the interaction thus might sponge the ubiquitylated effect on FOXO3 and facilitate MDM2-dependent ubiquitylation of p53, leading to cell apoptosis (34). Furthermore, circRNAs may participate in protein trafficking. The ecircRNA FECR1 binding to the promoter of its host gene FLI1 could recruit TET1 demethylase to induce DNA hypomethylation and activate transcription (41). The cooperative interactions between circRNAs and specific proteins may be one underappreciated mechanism of their modes of action.

### CircRNAs Undergo Translation

Though devoid of 5′ cap and 3′ polyadenylated tails structure, recent studies indicate that a subset of endogenous circRNAs can be translated in a cap-independent manner (35, 36, 42). Circ-ZNF609 was found to be associated with heavy polysomes and could be translated into a protein during muscle differentiation, albeit with a lower translation efficiency compared to its linear counterpart (42). In drosophila heads, a group of circRNAs was shown to be associated with translating ribosomes and one circRNA generated from the *muscleblind* (*mbl*) locus could synthesize a protein product as detected by mass spectrometry (35). The internal ribosome entry sites (IRESs) embedded within sequences or modification of N6-methyladenosine (m^6^A) was demonstrated to be capable to drive translation of circRNAs (36). However, the biological significance of circRNA translation remains largely unknown. It was shown that the efficiency of circRNA translation was altered under cellular stress (35, 36), indicating that cap-independent translation of circRNAs might be an adaptive mechanism under stress conditions.

## CircRNAs as Novel Biomarkers for Kidney Diseases

Mounting evidence suggests that circRNAs are abundant in a variety of body fluids, such as saliva, blood, urine, and exosomes secreted by most cell types (43–46). Furthermore, since circRNAs are exceptionally stable molecules, along with their cell-type-specific and tissue-specific expression patterns, their potentials as novel biomarkers in liquid biopsy have attracted an increasing interest of research. To date, results from many studies indicate that circRNAs could be represented as novel diagnostic and prognostic biomarkers in multiple human diseases, including cancer (47) and cardiovascular diseases (48). Preliminary attempts have been made to clarify the roles of circRNAs as biomarkers in kidney diseases in recent years. At present, this field of research are mainly focused on renal cell carcinoma (RCC), acute kidney injury (AKI), and glomerular diseases, including diabetic kidney disease (DKD), which are to be discussed in detailed (Table 1).

**Table 1:** Summary of candidate circRNAs as biomarkers in kidney diseases.

**CircRNA**	**Disease**	**Specimen**	**Expression change**	**Significance as biomarker**	**References**
circEGLN3	RCC	Kidney tissues	Up	As diagnostic biomarker of RCC; up-regulation predicts better prognosis	(49)
circRHOBTB3	RCC	Kidney tissues	Down	As diagnostic biomarker of RCC; down-regulation predicts poor prognosis	(49)
hsa_circ_0001451	RCC	Kidney tissues	Down	As diagnostic biomarker of RCC; down-regulation predicts poor prognosis	(50)
circPCNXL2	RCC	Kidney tissues	Up	Up-regulation predicts poor prognosis	(51)
circ-ABCB10	RCC	Kidney tissues	Up	Up-regulation predicts poor prognosis	(52)
hsa_circ_001895	RCC	Kidney tissues	Up	Up-regulation predicts poor prognosis	(53)
circ_001842	RCC	Kidney tissues	Up	Up-regulation predicts poor prognosis	(54)
circPRRC2A	RCC	Kidney tissues	Up	Up-regulation predicts poor prognosis	(55)
circ-EGLN3	RCC	Kidney tissues	Up	Up-regulation predicts poor prognosis	(56)
circ_0001368	RCC	Kidney tissues	Down	Down-regulation predicts poor prognosis	(57)
cRAPGEF5	RCC	Kidney tissues	Down	Down-regulation predicts poor prognosis	(58)
ciRs-126	AKI	Blood	Up	Up-regulation predicts poor 28-day survival	(59)
hsa_circ_0001334	acute kidney rejection	Urine	Up	As diagnostic biomarker of acute rejection; up-regulation predicts poor 1-year graft function	(45)
circ_101319	IMN	Blood	Up	As diagnostic biomarker of IMN	(60)
circ_002453	LN	Blood	Up	As diagnostic biomarker of LN	(61)
hsa_circ_0123190	LN	Blood	Down	As diagnostic biomarker of LN	(62)

*RCC, renal cell carcinoma; AKI, acute kidney injury; IMN, idiopathic membranous nephropathy; LN, lupus nephritis*.

### CircRNAs and RCC

RCC is the most common type of kidney neoplasm, accounting for 85–90% of adult renal malignancies (63). Early diagnosis of RCC and timely identification of post-operative recurrence and metastasis are crucial for improving the outcome of patients with RCC. Reliable biomarkers to fulfill these clinical expectations are urgently needed. Recently, dysregulated circRNA expression profiles have been reported in RCC. Five hundred forty-two circRNAs were identified as differentially expressed by using RNA microarray data from online RCC database (64). Among these, 324 circRNAs were down-regulated, whereas 218 were up-regulated in the ccRCC group. Another study performed a genome-wide screening of dysregulated circRNAs using 7 matched clear cell RCC (ccRCC) tissue samples by microarray analysis and 78 circRNAs were up-regulated while 91 were down-regulated in malignant tissues compared to adjacent normal samples (49). The expressions of three selected circRNAs (circEGLN3, circNOX4, and circRHOBTB3) were validated by quantitative reverse transcriptase-polymerase chain reaction (qRT-PCR) assays. By performing receiver-operating characteristics curve (ROC) analysis, these circRNAs demonstrated excellent diagnostic values to diagnose ccRCC, with the AUC values of circNOX4, circRHOBTB3, and circEGLN3 as 0.81, 0.82, and 0.98, respectively. Moreover, the predictive accuracy of a clinical model based on clinicopathological variables of ccRCC was significantly improved by including the expression signature of these three circRNAs. Another study found that the expression of hsa_circ_0001451 was shown to be significantly decreased in ccRCC tissues and correlated with tumor staging and metastasis (50). The AUC-ROC value of this circRNA for diagnosis of ccRCC was 0.704 and regression analysis disclosed that the level of hsa_circ_0001451 was an independent predictor for overall survival of ccRCC patients, supporting this circRNA as a reliable diagnostic and predictive indicator of ccRCC. Other circRNAs that have been reported to predict prognosis for RCC included circPCNXL2, circ-ABCB10, hsa_circ_001895, circ_001842, circPRRC2A, circ-EGLN3, circ_0001368, and cRAPGEF5 (51–58). The former six circRNAs were all found to be up-regulated in tumor tissues and associated with poor clinical outcomes in ccRCC patients. The latter two was shown to be significantly reduced in RCC samples. The decreased level of cRAPGEF5 was negatively correlated with tumor growth and metastasis and shown to be an independent factor for poor prognosis in RCC patients. These findings supported the roles of circRNAs as novel potential biomarkers for the diagnosis and outcome prediction of RCC.

### CircRNAs and AKI

AKI is an increasingly common clinical syndrome characterized by the rapid decline in kidney function and has a relatively high mortality rate with no specific treatment beyond supportive care. Exploration of novel biomarkers besides serum creatinine and urine output for early diagnosis of AKI remains an area of utmost interest. In recent years, a large number of circRNAs have been found to be differentially expressed in AKI animal models induced by ischemia and reperfusion (I/R) or cisplatin treatment (65, 66). Interestingly, the dysregulated circRNA profiles following I/R treatment could be restored by pre-treatment with the drug losartan accompanied by improvements in the functional and histological indicators of kidney injury (65), implying that circRNAs might play a role in AKI development and mediate the renoprotective effect of losartan. As AKI is a severe complication in critically ill patients in intensive care unit (ICU), a genome-wide circRNA expression analysis was performed using RNA isolated from whole blood of ICU patients and revealed that ciRs-126 (circRNA sponge of miR-126) was significantly increased in AKI patients compared to healthy and disease controls (59). Further Cox regression and Kaplan Meier curve analysis identified ciRs-126 as a strong independent risk factor for 4-week-survival. By ROC curve analysis, ciRs-126 levels yielded an AUC value of 0.92 with 91% sensitivity and 74% specificity. Though this is a single-center experience with relatively small sample size, these data suggest ciRs-126 might act as a useful biomarker for predicting the outcome of AKI patients in ICU settings. In another study conducted by the same study group, the global expression profile of urinary circRNAs was identified in patients with acute renal allograft rejection and hsa_circ_0001334 in urine was shown to be up-regulated in patients with acute rejection compared to controls and normalized following successful anti-rejection therapy (45). Importantly, the elevated levels of this circRNA could be measured at subclinical time points of rejection while there are no elevations in serum creatinine.Hsa_circ_0001334 yielded an AUC value of 0.85 with a sensitivity of 70.11% and specificity of 92.31% in diagnosing acute rejection. In addition, the increased expression of hsa_circ_0001334 was positively associated with decline of kidney function 1 year after transplantation. Thus, urinary hsa_circ_0001334 might serve as a novel non-invasive marker of acute kidney rejection and predictor of graft function.

### CircRNAs and Glomerular Diseases

Glomerular diseases refer to a large group of diseases that injuries mainly involve bilateral glomeruli, which can be classified into three categories: primary, secondary, and inherited glomerular diseases. Glomerular diseases have a very high morbidity worldwide and remain the primary cause of chronic kidney disease (CKD) and end-stage renal disease (ESRD), which pose huge burden on society and economy. At present, the diagnosis of glomerular diseases mainly depends on invasive kidney biopsy and reliable indicators for predicting clinical outcomes of specific glomerular diseases are lacking. Recently, increasing studies have focused on the expression and roles of circRNAs in the diagnosis and prognosis prediction of various glomerular diseases.

Diabetic kidney disease (DKD) is one of the most frequent complications of diabetes mellitus and the leading cause of CKD worldwide, which is pathologically characterized by mesangial cells (MCs) proliferation, extracellular matrix (ECM) accumulation, and basement membrane thickening. Nevertheless, there are few reports evaluating the roles of circRNAs as biomarkers in DKD up to date. One study investigated the differentially expressed (DE) circRNA profiles in the *db*/*db* mouse model by microarray analysis (67). Another study performed high-throughput circRNA sequencing using the same model and identified 40 DE circRNAs, among which 18 were up-regulated and 22 were down-regulated in diabetic mouse kidneys (68). Circ_0080425 was found to be significantly increased in kidneys of streptozotocin-treated diabetic model and positively correlated with the severity of pathological abnormalities (69). Another study constructed an *in vitro* high glucose (HG)-treated glomerular endothelial cells (GECs) model and obtained exosomal circRNA profiles secreted by GECs using high-throughput sequencing. Compared to normal glucose (NG)-treated GEC exosomes, 217 circRNAs were significantly down-regulated while 484 were up-regulated in HG-treated GEC exosomes (70). The level of circ_DLGAP4 was also shown to be increased in exosomes isolated from HG-treated MCs (71). Moreover, its expression was consistently elevated in DKD rat models, DKD patients, and further increased in DKD patients with macroalbuminuria, suggesting it might correlate with DKD progression. These preliminary studies suggest that dysregulated circRNAs, especially those from exosomes, are potential biomarkers for DKD. However, further studies are needed to verify these DE circRNAs as reliable biomarkers of DKD. Furthermore, there is a lack of research to conduct global screening for biomarkers using samples from DKD patients. It would also be interesting to search for circRNAs that could distinguish true DKD patients from those with non-DKD (NDKD).

IgA nephropathy (IgAN) is the most common type of primary glomerulonephritis worldwide. To date, only two studies have analyzed the dysregulated circRNAs profiles in IgAN patients (72, 73). The first study conducted circRNA sequencing using RNA isolated from peripheral blood mononuclear cells (PBMCs) of three pairs of IgAN patients and healthy controls (72). A total of 145 circRNAs were identified as differentially expressed, among which 112 circRNAs were up-regulated while 33 were down-regulated in IgAN group compared to controls. A recent study investigated the circRNAs profiles in urinary exosomes from five pairs of IgAN patients and healthy controls by high-throughput RNA sequencing (73). In total, 1,322 circRNAs were detected in the urinary exosomes and 476 were aberrantly expressed, including 450 up-regulated and 26 down-regulated circRNAs. These two studies are relatively preliminary with small sample sizes. The roles of these dysregulated circRNAs as biomarkers in IgAN warrant further exploration.

Membranous nephropathy (MN) is another common type of glomerulopathy with increasing frequency over the past decades (74). A preliminary study profiled the expression of circRNAs in exosomes from both serum and urine in patients with idiopathic membranous nephropathy (IMN) and identified 89 DE circRNAs in serum exosomes and 60 DE circRNAs in urinary exosomes (75). Another study conducted microarray analysis to identify circRNA profiles in the peripheral blood of IMN patients and showed that 955 circRNAs were differentially expressed, of which 645 were up-regulated and 310 were down-regulated (60). The increased expression of circ_101319 in the IMN group was validated by qRT-PCR. ROC curve analysis revealed that the AUC value for circ_101319 to diagnose IMN was 0.89, with a sensitivity of 93.33% and specificity of 70.00%, suggesting circ_101319 might act as a reliable biomarker for the diagnosis of IMN. As there is increasing focus on the role of anti-PLA2R antibody as a key biomarker in the diagnosis and monitoring of IMN (76), it would be intriguing to search for candidate circRNAs that could enhance the diagnostic and predictive power of the anti-PLA2R antibody.

Lupus nephritis (LN) is the most common complication of systemic lupus erythematosus (SLE) and 10–30% of LN patients will progress to ESRD (77). Initially, several studies used microarray or high-throughput RNA sequencing to screen the circRNAs profiles in peripheral blood of SLE patients (78, 79). One study identified hsa_circ_0000479 was significantly increased in SLE patients compared to controls and its high expression was associated with low albumin levels and positive urine protein (79). Another study profiled the circRNAs expression by RNA sequencing using renal biopsy tissues from LN patients (80). Among dysregulated circRNAs, circHLA-C positively correlated with proteinuria, serum creatinine, percentage of crescentic glomeruli, and renal activity index. Subsequent studies made further efforts to explore the diagnostic values of circRNAs in LN. One study showed that plasma circRNA_002453 was significantly up-regulated in patients with LN compared to SLE patients without LN, rheumatoid arthritis (RA) patients, and healthy controls (61). Interestingly, the level of circRNA_002453 was positively associated with proteinuria and renal SLEDAI score but not with systemic activity makers. ROC analysis revealed that circRNA_002453 yielded an AUC value of 0.906 to diagnose LN. Another study identified hsa_circ_0123190 was down-regulated in both renal tissues and peripheral blood of LN patients (62). There was no significant association between the tissue levels of hsa_circ_0123190 and clinical parameters, while its expression in blood was negatively correlated with serum creatinine, and the AUC-ROC value diagnosing LN was 0.900. Results of these studies suggested that circRNA_002453 and hsa_circ_0123190 could be reliable biomarkers for the diagnosis of LN.

## CircRNAs as Therapeutic Targets for Kidney Diseases

Although lots of efforts have been made in kidney diseases field, the pathogenesis of most renal disorders remains largely unclear, which hinders the development of specific therapeutic strategies to treat these diseases. Since circRNAs could regulate gene expression and are differentially expressed under pathological conditions, they are probably biologically functional in diseases. In fact, current data indicate that circRNAs are involved in the pathogenesis and progression of cancer (16), cardiovascular diseases (17), and neurological disorders (19). Recently, studies have found that a great number of dysregulated circRNAs expressed by kidney resident cells contribute to the initiation and development of multiple kidney diseases, mostly reported in DKD, RCC, as well as other types of renal disorders (22, 23). Mechanistically, the majority of these circRNAs were shown to exert their biological functions by acting as miRNA sponges. Although most of these findings are from *in vitro* cell culture experiments, these studies provide novel avenues to elucidate the mechanisms underlying kidney diseases and make circRNAs novel promising therapeutic targets in this realm.

### CircRNAs and RCC

In recent years, a growing number of studies have shown that circRNAs play critical roles in the tumorigenesis and progression of RCC (Table 2). These circRNAs are involved in various processes of RCC development, including cell proliferation, migration, invasion, apoptosis, and EMT. The majority of circRNAs identified to date are shown to exert oncogenic effects, and the most common studied mechanism is to act as miRNA sponges. For instance, circPCNXL2 was highly expressed in ccRCC tissues compared to adjacent non-tumor tissues (51). Functionally, circPCNXL2 inhibition markedly repressed RCC cells proliferation and invasion, suggesting it serve as an oncogenic circRNA in RCC progression. Mechanistically, circPCNXL2 functioned as a sponge for miR-153 to modulate the expression of its target gene ZEB2, which is a known positive driver in tumor progression. Another study reported that the high expression of circ_000926 facilitated the development and progression of RCC by sponging miR-411 to up-regulate CDH2, which is a marker of EMT and contributor to RCC aggressiveness (81). A recent study found a novel circRNA (circPRRC2A) promoted angiogenesis and metastasis of RCC (55). Further mechanistic experiments revealed that circPRRC2A could directly bind to miR-514a-5p and miR-6776-5p to manipulate the control of TRPM3-induced EMT. On the other hand, several circRNAs were shown to function as tumor suppressors in RCC. Circ-AKT3 was stably decreased in both ccRCC cell lines and tumor tissues (82). Restoration of circ-AKT3 inhibited cell migration and invasion by acting as a sponge of miR-296-3p and up-regulating E-cadherin expression, supporting a protective role of circ-AKT3 in ccRCC metastasis. Like circ-AKT3, cRAPGEF5 was significantly down-regulated in RCC (58). Functional assays demonstrated that cRAPGEF5 suppressed tumor growth and metastasis of RCC by sponging oncogenic miR-27a-3p, which targets the suppressor gene TXNIP. Circ_0001368 was also identified as a novel anti-tumor RNA via negatively regulating the miR-492/LATS2 axis (57). Several other circRNA-miRNA-mRNA interaction cascades have been reported in RCC, most of which were found with tumor promotive effects, including circ_001895/miR-296-5p/SOX12 (53), circ_001842/miR-502-5p/SLC39A14 (54), circ-EGLN3/miR-1299/IRF7 (56), circ_0039569/miR-34a-5p/CCL22 (83), circ-ZNF609/miR-138-5p/FOXP4 (84), and circ_0054537/miR-130a-3p/cMet (85). Intriguingly, several circRNAs have been reported to work as miRNA reservoirs to regulate RCC development (86, 87). CircHIAT1 was involved in the androgen receptor (AR)-driven RCC progression by serving as a miRNA reservoir to increase the stability and availability of miR-195-5p/29a-3p/29c-3p, leading to the suppression of AR-enhanced ccRCC migration and invasion (86). CircATP2B1 was found to be repressed by estrogen receptor beta (ERβ), which functions as an oncogene in RCC metastasis (87). Overexpression of circATP2B1 could increase miR-204-3p stability by acting as a miRNA reservoir to partially reverse ERβ-promoted ccRCC invasion. In addition to the manipulation of hub genes, recent evidence has revealed that the circRNA-miRNA network regulate RCC progression through tumor-associated signaling pathways. For instance, circ-0072309 played anti-tumor roles by sponging miR-100 to block the phosphoinositide 3-kinase (PI3K)/protein kinase B (AKT)/mammalian target of rapamycin (mTOR) pathway signaling cascades, which are pivotal pathways in RCC development (88). Recently, an interesting study attempted to explore the roles of circRNAs in chemotherapy resistance of RCC patients (89). This study revealed that hsa_circ_0035483 was highly expressed in RCC and facilitated gemcitabine resistance and tumor growth by modulating the hsa-miR-335/CCNB1 axis. Importantly, silencing hsa_circ_0035483 enhanced gemcitabine sensitivity and repressed tumor growth *in vivo*, suggesting hsa_circ_0035483 could be a promising therapeutic target of gemcitabine resistance in RCC treatment. *In vivo* manipulation of circRNAs expression is vital to clarify their functional relevance. In fact, many studies have already explored the effect of interfering with circRNAs expression on RCC growth and metastasis *in vivo* (51, 53–55, 57, 58, 81, 82, 86, 87). These studies mainly adopted the strategy of injecting RCC cell lines stably overexpressing or knocking down specific circRNAs, most of which were achieved by transfection with lentivirus plasmids, into nude mice. One study performed subcutaneous inoculations of RCC cell lines stably depleting circ_000926 by transfection with siRNA against circ_000926 and observed inhibitory effects on tumor growth (81). Findings from *in vivo* experiments further confirmed the potential of dysregulated circRNAs as therapeutic targets for RCC. Nevertheless, more efforts are needed to fully evaluated the safety and specificity of these ectopic circRNA intervention strategies.

**Table 2:** Summary of candidate circRNAs as therapeutic targets in renal cell carcinoma (RCC).

**CircRNA**	**Expression change**	**Function**	**Target miRNA**	**MiRNAs target genes/pathways**	**References**
hsa_circ_0001451	Down	Inhibiting RCC cell proliferation and promoting apoptosis	ND	ND	(50)
circPCNXL2	Up	Promoting RCC cell proliferation, invasion, and tumor growth	miR-153	ZEB2	(51)
circ-ABCB10	Up	Promoting RCC cell proliferation and inhibiting apoptosis	ND	ND	(52)
hsa_circ_001895	Up	Promoting RCC cell proliferation, migration, invasion, and inhibiting apoptosis	miR-296-5p	SOX12	(53)
circ_001842	Up	Promoting RCC cell proliferation, migration, invasion, EMT, and tumor growth	miR-502-5p	SLC39A14	(54)
circPRRC2A	Up	Promoting RCC cell proliferation, migration, invasion, angiogenesis, EMT, tumor growth, and metastasis	miR-514a-5p, miR-6776-5p	TRPM3	(55)
circ-EGLN3	Up	Promoting RCC cell proliferation, migration, invasion, and inhibiting apoptosis	miR-1299	IRF7	(56)
circ_0001368	Down	Inhibiting RCC cell proliferation and invasion	miR-492	LATS2	(57)
cRAPGEF5	Down	Inhibiting RCC cell proliferation, migration, invasion, and tumor growth and metastasis	miR-27a-3p	TXNIP	(58)
circ_000926	Up	Promoting RCC cell proliferation, migration, invasion, EMT, and tumor growth	miR-411	CDH2	(81)
circ-AKT3	Down	Inhibiting RCC cell migration, invasion, and tumor metastasis	miR-296-3p	E-cadherin	(82)
circ-0039569	Up	Promoting RCC cell proliferation, migration, and invasion	miR-34a-5p	CCL22	(83)
circ-ZNF609	Up	Promotes RCC cell proliferation and invasion	miR-138-5p	FOXP4	(84)
hsa_circ_0054537	Up	Promoting RCC cell proliferation, migration, and inhibiting apoptosis	miR-130a-3p	c-Met	(85)
circHIAT1	Down	miRNA reservoir; inhibiting RCC cell migration and invasion	miR-195-5p/29a-3p/29c-3p	CDC42	(86)
circATP2B1	Down	miRNA reservoir; inhibiting RCC cell invasion	miR-204-3p	FN1	(87)
hsa-circ-0072309	Down	Inhibiting RCC cell proliferation, migration, invasion, and promoting apoptosis	miR-100	PI3K/AKT, mTOR	(88)
hsa_circ_0035483	Up	Promoting autophagy and the resistance of RCC to gemcitabine	hsa-miR-335	CCNB1	(89)

*RCC, renal cell carcinoma; ND, not determined; EMT, epithelial–mesenchymal transition*.

### CircRNAs and DKD

Hyperglycemia is an essential contributor for the pathogenesis of DKD and additional mechanisms, such as oxidative stress, inflammation, participate in the development of DKD as well (90). Almost all types of kidney resident cells are affected and altered under the diabetic milieu. The studies evaluating the functional relevance of circRNAs in DKD have just begun to be increasing in recent 2 years (Table 3). Most adopted *in vitro* cell culture models induced by HG stimulation, among which mesangial cell lines were the most frequently used. MCs proliferation, ECM production, and fibrosis were the most frequently studied biological phenomena, and most circRNAs identified to date were found to be increasingly expressed in DKD. For instance, the first study regarding circRNA in DKD showed that circRNA_15698 was significantly up-regulated in both HG-treated MCs and DKD mice (67). CircRNA_15698 knockdown suppressed the synthesis of fibrosis-related proteins in HG-treated MCs, suggesting this circRNA positively regulate the fibrotic process. Further experiments revealed that circRNA_15698 functioned as a sponge for miR-185 and subsequently increased the expression of its target gene TGF-β1, one of the master regulators in fibrosis, leading to enhanced ECM accumulation in DKD. The second relevant study found circLRP6 was highly expressed in HG-treated MCs and could promote cell proliferation, oxidative stress, ECM accumulation, and inflammation (91). This circRNA exerted its functions by sponging miR-205 to activate the classical pro-inflammatory TLR4/NF-κB pathway. The elevated expression of circ_0080425 exerted positive effect on cell proliferation and fibrosis in MCs via sponging miR-24-3p to up-regulate FGF11 (69). Circ_0000491 aggravated ECM and fibrosis-associated protein synthesis through suppressing miR-101b which targets TGFβRI (68). Circ_0123996 was able to promote MCs proliferation and fibrosis through functioning as the sponge for miR-149-5p and inducing Bach1 expression (92). Circ_00037128/miR-17-3p/AKT3 axis also facilitated DKD progression via modulating MCs proliferation and fibrosis (93). On the contrary, another two circRNAs, circ-AKT3 and circ_LARP4, were found to be down-regulated in HG-stimulated MCs model (94, 95). Circ-AKT3 inhibited ECM accumulation via modulating miR-296-3p/E-cadherin signals, while circ_LARP4 overexpression could repress MCs proliferation and fibrosis but increase cell apoptosis by sponging miR-424. As discussed above, the circ-AKT3/miR-296-3p/E-cadherin axis also plays a role in suppressing renal cancer metastasis (82), implying that circRNA-mediated regulatory networks might function under diverse disease conditions. Though current data was mainly derived from MCs, circRNAs may also play a role in other kidney cell types, such as tubular epithelial cells (TECs) and podocytes. The expression of several circRNAs, including hsa_circ_0003928, circ_WBSCR17, circACTR2, and circEIF4G2, were shown to be increased by HG stimulation in TECs model (96–99). Interference of hsa_circ_0003928 alleviated HG-induced secretion of inflammatory cytokines and repressed apoptosis in TECs by sponging miR-151-3p partly through regulating Anxa2, suggesting this circRNAs could positively modulate inflammation and apoptosis (96). Circ_WBSCR17 aggravated HG-induced inflammatory responses and fibrosis in HK-2 cells by activating SOX6 via targeting miR-185-5p (97). CircACTR2 was shown to promote HG-induced pyroptosis, inflammation, and fibrosis in TECs (98), yet the underlying mechanisms remain to be defined. CircEIF4G2 positively regulated the synthesis of fibrosis-related proteins in HG-induced TECs via the miR-218/SERBP1 pathway (99). One circRNA, circ_0000285, was found to be significantly increased in podocytes treated with HG as well as in DKD mouse kidneys (100). Up-regulation of circ_0000285 contributed to the development of DKD by triggering podocyte injuries through sponging miR-654-3p and activating MAPK6. Interestingly, results of several studies in DKD indicated that different circRNAs could act as the same miRNA's sponge. For example, miR-143 was confirmed to be targeted by both circ_DLGAP4 and circ_0000064 in MCs, whose increased expression consistently promoted cell proliferation and fibrosis (71, 101). As mentioned above, circ_WBSCR17 functioned through sponging miR-185-5p in TECs. Another study revealed that circHIPK3 could target miR-185-5p in MCs (102). These two circRNAs exhibited promotive functions on fibrosis both through negatively regulating miR-185-5p, in tubulointerstitial and glomerular compartments, respectively. Nevertheless, circHIPK3 was demonstrated to protect TECs from HG-induced toxicity via sponging miR-326/miR-487a-3p in another report (103), suggesting the same circRNA could regulate different miRNAs in different cell types and exert diverse effects. At present, there is a lack of research on *in vivo* manipulation of circRNA expression in DKD animal models. A recent study attempted to interfere with the expression of circRNA_010383 *in vivo* (104). They used a well-established ultrasound-microbubble-mediated gene transfer technique to specifically deliver the circRNA_010383 expression plasmid into the kidneys. By intermittent ultrasound-mediated circRNA_010383 transfer, its expression level was markedly restored in diabetic mouse kidneys, leading to amelioration of renal fibrosis. These studies suggest that circRNAs may play vital roles in the pathogenesis of DKD and can act as potential therapeutic targets for DKD, which undoubtedly requires further explorations.

**Table 3:** Summary of candidate circRNAs as therapeutic targets in diabetic kidney disease (DKD).

**CircRNA**	**Expression change**	**Function**	**Target miRNA**	**MiRNAs target genes/pathways**	**References**
circRNA_15698	Up	Promoting ECM-related proteins synthesis	miR-185	TGF-β1	(67)
circ_0000491	Up	Promoting ECM-related proteins synthesis	miR-101b	TGFβRI	(68)
circ_0080425	Up	Promoting MCs proliferation and fibrosis	miR-24-3p	FGF11	(69)
circ_DLGAP4	Up	Promoting MCs proliferation and fibrosis	miR-143	ERBB3/NF-κB/MMP-2	(71)
circLRP6	Up	Promoting MCs proliferation, oxidative stress, ECM accumulation, and inflammation	miR-205	HMGB1/TLR4/NF-κB pathway	(91)
circ_0123996	Up	Promoting MCs proliferation and fibrosis	miR-149-5p	Bach1	(92)
circ_0037128	Up	Promoting MCs proliferation and fibrosis	miR-17-3p	AKT3	(93)
circ-AKT3	Down	Inhibiting ECM-related proteins synthesis	miR-296-3p	E-cadherin	(94)
circ_LARP4	Down	Inhibiting MCs proliferation and fibrosis and promoting MCs apoptosis	miR-424	ND	(95)
hsa_circ_0003928	Up	Promoting TECc apoptosis and inflammation	miR-151-3p	Anxa2	(96)
circ_WBSCR17	Up	Promoting TECs apoptosis, inflammation, and fibrosis	miR-185-5p	SOX6	(97)
circACTR2	Up	Promoting TECs pyroptosis, inflammation, and fibrosis	ND	ND	(98)
circEIF4G2	Up	Promoting fibrotic markers synthesis	miR-218	SERBP1	(99)
circ_0000285	Up	Promoting podocyte injury	miR-654-3p	MAPK6	(100)
circ_0000064	Up	Promoting MCs proliferation and fibrosis	miR-143	ND	(101)
circHIPK3	Up	Promoting MCs proliferation and fibrosis	miR-185	ND	(102)
circHIPK3	Down	Promoting TECs proliferation and inhibiting apoptosis	miR-326/miR-487a-3p	SIRT1	(103)
circRNA_010383	Down	Inhibiting ECM-related proteins synthesis	miR-135a	TRPC1	(104)

*ECM, extracellular matrix; MCs, mesangial cells; ND, not determined; TECs, tubular epithelial cells*.

### CircRNAs and AKI

Although accumulating evidence suggests that circRNAs are aberrantly expressed in AKI animal models and clinical samples (59, 66), there are few studies to characterize their roles in the pathogenesis of AKI. One study found a novel circRNA, circular antisense non-coding RNA in the INK4 locus (cANRIL), was induced by lipopolysaccharides (LPS) treatment in HK-2 cells (105). Silencing cANRIL alleviated LPS-induced inflammatory injuries and oxidative stress in HK-2 cells by blocking NF-κB and c-Jun N-terminal kinase (JNK)/p38 pathways via increasing miR-9 expression. Another study performed high-throughput RNA sequencing using renal tubular tissues from cisplatin-induced AKI mice models and identified a novel circRNA (circ-0114427) in human by comparing homologous genes between mouse and human (106). Circ-0114427 was remarkably increased in several AKI cell models and could exert anti-inflammatory effects in early stages of AKI development. Mechanistically, circ-0114427 directly sponged miR-494 to up-regulate the expression of activating transcription factor 3 (ATF3) and then inhibited the secretion of downstream cytokine IL-6. These findings identified a novel circ-0114427/miR-494/ATF3/IL-6 regulatory axis in AKI progression. Different from the roles in DKD and RCC, circ-AKT3 was shown to promote cell apoptosis and enhance oxidative stress in AKI induced by I/R treatment (107). Results of this study revealed that circ-AKT3 could activate the Wnt/β-catenin signal via functioning as a sponge for a different miRNA (miR-144-5p), implying that the same circRNA could regulate different miRNAs to exert multifaceted functions under diverse pathological circumstances. Of note, the research regarding the roles of circRNAs in AKI is still in the preliminary stage, and more in-depth research is warranted in the future.

### CircRNAs and LN

As discussed above, many circRNAs were found to be differentially expressed in patients with SLE or LN. These dysregulated circRNAs may play roles in the pathogenesis and progression of SLE and LN. However, only two studies have made initial attempts to explore the functions of circRNAs in LN at present. One study identified that circHLA-C was the most significantly increased circRNA in LN and displayed a tendency of negative correlation with miR-150 (80). Further bioinformatic analysis revealed circHLA-C harbored a perfect match binding sequence for miR-150. Since miR-150 was previously reported to promote renal fibrosis in LN, these results suggest that circHLA-C might participate in the development of LN by sponging miR-150. The other study found hsa_circ_0123190 was down-regulated in LN and could serve as a sponge for hsa-miR-483-3p, which targets apelin receptor (APLNR) (62). APLNR has been shown to be involved in renal fibrosis by acting on TGF-β1 and its expression was associated with chronicity index (CI) of LN. Therefore, hsa_circ_0123190 might contribute to renal fibrosis in LN by modulating the hsa-miR-483-3p/APLNR/TGF-β1 axis. Nevertheless, results of these two studies are relatively preliminary. Future investigations by gain-of-function and loss-of-function *in vitro* and *in vivo* experiments are needed to clarify the functional roles of candidate circRNAs as potential therapeutic targets in LN.

### CircRNAs and Other Kidney Diseases

Focal and segmental glomerulosclerosis (FSGS), a common histopathological lesion which is characterized by segmental glomerular scarring, is one of the leading causes of adult nephrotic syndrome and ESRD worldwide. One study found that circZNF609 was up-regulated in both adriamycin-induced FSGS mouse kidneys and bovine serum albumin (BSA)-treated HK-2 cells, while miR-615-5p showed the opposite trend (108). The intra-renal expression of circZNF609 was positively correlated while miR-615-5p was negatively correlated with podocyte injury and renal fibrosis. Moreover, perfect match sequences between circZNF609 and miR-615-5p were predicted by bioinformatics tools. These results suggest circZNF609 might be involved in the pathogenesis of FSGS by targeting miR-615-5p. However, these preliminary findings are simply correlation data of expression changes, which undoubtedly need to be validated in future functional experiments.

Hypertension is a common chronic disease with a high prevalence in the general population which frequently produces adverse effects on certain target organs, including heart, blood vessels, and kidney. Recent studies indicate many circRNAs were aberrantly expressed in blood samples of hypertensive patients or kidneys of hypertensive models (109–111). Among them, circNr1h4 derived from the Nr1h4 gene was significantly decreased in hypertensive mouse kidneys (111). Mechanistic investigations revealed that circNr1h4 modulated renal injury by sponging miR-155-5p to regulate its target gene fatty acid reductase 1 (Far1). These results provide novel insights into underlying mechanisms of hypertension and hypertensive nephropathy, which require further investigations.

Vascular calcification (VC) is one of the common complications in CKD patients. One study using RNA sequencing identified that a large number of circRNAs changed significantly in a cellular model of VC (112). Among them, circSamd4a played an anti-calcification role in VC as overexpressing it could reduce VC. Mechanistically, circSamd4a acted as a sponge for miR-125a-3p and miR-483-5p to regulate downstream genes related to calcium modulation. This study provides novel mechanisms for the development of VC and circSamd4a may serve as a promising therapeutic target for VC in CKD patients since it is conserved in humans.

## Conclusions and Future Perspectives

Recent advances in the circRNA research field have uncovered their diversified functions in health and disease. In the past 5 years, increasing endeavors have identified numerous circRNAs involved in the pathogenesis and progression of various kidney diseases. These putative circRNAs function mainly through circRNA-miRNA-mRNA networks, expanding our understanding of regulatory mechanisms underlying kidney disorders. In addition, owing to their impressive stability, circRNAs have been validated as reliable biomarkers for diagnosis and prognosis prediction of multiple kidney diseases. Results of these investigations support that circRNAs possess the promising potential as both biomarkers and therapeutic targets in the kidney realm.

However, we have to admit that there are shortcomings regarding current research and many challenges remain to be overcome in this field. Firstly, most studies investigating circRNAs as biomarkers are from single-center, with small sample sizes, and lack of independent external cohort validation. Larger independent cohorts from multi-center settings are highly desirable to validate candidate circRNAs as promising biomarkers. In addition, there are few studies searching for biomarkers to monitor treatment efficacy or predict relapse of glomerular diseases, including nephritic syndrome, lupus nephritis, and vasculitis-associated renal lesions. Exosomal circRNAs in blood and urine are promising biomarkers for non-invasive liquid biopsy and more standardized techniques are desired to reliably detect these exosomal circRNAs. Secondly, the vast majority of aberrantly expressed circRNAs have not been studied functionally and those possibly as hub genes in signaling pathways underlying kidney diseases remain to be identified. To date, most studies on circRNAs are focused on their function as miRNA sponges. It is likely that those dysregulated circRNAs may function through other regulatory mechanisms, such as transcription regulation or acting as RBP sponges, which warrants further research. Last but not the least, the biological functions and therapeutic potential of candidate circRNAs need to be verified in animal models, including non-human primates. There is still lack of ways to specifically and efficiently deliver circRNAs into recipient cells *in vivo* and whether interfering specific circRNAs expression would produce off-target effects remains uncharacterized. Nevertheless, it is becoming clear that increasing exploration into the potential roles of circRNAs will extend our understanding of kidney diseases and hopefully will become an intense area of research in the near future.

## Author Contributions

JY and DX wrote the manuscript. NH drew the figure and tables. QZ supervised the manuscript. All authors contributed to article design, revision, read, and approved the submitted version.

## Funding

This study was supported by program from Guangdong Basic and Applied Basic Research Foundation (2020A1515010247); Guangzhou Science and Technology Innovation Commission (201806010123); Kelin Young Talents Program of the First Affiliated Hospital of Sun Yat-sen University (Y50179); National Key R&D Program of China (2016YFC0906101); Operational Grant of Guangdong Provincial Key Laboratory (2017B030314019); Guangdong Provincial Programme of Science and Technology (2017A050503003 and 2017B020227006); Guangzhou Municipal Programme of Science and Technology (201704020167); Natural Science Foundation of Guangdong Province, China (Grant No. 2017A030310199); and National Natural Science Foundation Grant (82170732).

## Conflict of Interest

The authors declare that the research was conducted in the absence of any commercial or financial relationships that could be construed as a potential conflict of interest.

## Publisher's Note

All claims expressed in this article are solely those of the authors and do not necessarily represent those of their affiliated organizations, or those of the publisher, the editors and the reviewers. Any product that may be evaluated in this article, or claim that may be made by its manufacturer, is not guaranteed or endorsed by the publisher.
